# Epidemiological distribution of primary central nervous system tumors in the Western Province of Saudi Arabia: a local registry from neuroscience-affiliated centers

**DOI:** 10.4178/epih.e2021037

**Published:** 2021-05-23

**Authors:** Maher Kurdi, Nadeem Shafique Butt, Saleh Baeesa, Badrah Alghamdi, Yazid Maghrabi, Anas Bardeesi, Rothaina Saeedi, Ahmed I. Lary

**Affiliations:** 1 Department of Pathology, Faculty of Medicine, King Abdulaziz University, Rabigh, Kingdom of Saudi Arabia; 2 Department of Family and Community Medicine, Faculty of Medicine, King Abdulaziz University, Jeddah, Kingdom of Saudi Arabia; 3 Division of Neurosurgery, Department of Surgery, Faculty of Medicine, King Abdulaziz University, Jeddah, Kingdom of Saudi Arabia; 4 Department of Physiology, Faculty of Medicine, King Abdulaziz University, Jeddah, Kingdom of Saudi Arabia; 5 Department of Neuroscience, King Faisal Specialist Hospital and Research Center, Jeddah, Kingdom of Saudi Arabia; 6 Section of Neurosurgery, Department of Surgery, King Abdulaziz Medical City, Jeddah, Kingdom of Saudi Arabia

**Keywords:** Central nervous system tumour, Brain tumour, Epidemiology, Incidence, Saudi Arabia, Asia

## Abstract

**OBJECTIVES:**

Central nervous system (CNS) tumors are a major and growing global healthcare challenge. Western Saudi Arabia has an inconsistent data registry; therefore, the epidemiology of CNS tumors is unclear across the country. This study is aimed to assemble the epidemiological matrix of CNS tumors in the Western Province of Saudi Arabia.

**METHODS:**

A retrospective analysis was performed using clinical data obtained from 3 neuroscience centers in Western Saudi Arabia in the period 2014-2019. The sample size included 663 adult and pediatric cases from the local and expatriate populations diagnosed with CNS tumors. The distributions of age, sex, clinical presentation, tumor location, type of surgery, histological subtype, genetic characteristics, and recurrence rate were explored.

**RESULTS:**

The analysis included 500 adult cases and 163 pediatric cases up to 18 years of age with a male-to-female ratio of 1.16. The mean age at diagnosis was 38.0±22.6 years. The supratentorium was the most common location (n=515, 77.7%). Most patients presented with headache (n=298, 44.9%), followed by a focal neurological deficit (19.9%). The most common primary CNS tumor was glioblastoma (n=234, 35.3%), followed by meningioma (n=100, 15.1%). The recurrence rate after surgery was estimated to be 40.9% among all CNS tumors.

**CONCLUSIONS:**

This is the first tumor registry of Western Province of Saudi Arabia that describes the distribution of primary CNS tumors and highlights their epidemiological matrix. Several incidence trends in terms of histological type, age group, sex, location, and recurrence were determined, and some genetic characteristics were recognized.

## INTRODUCTION

Systemic tumors, particularly cancers, are responsible for more than 9 million deaths worldwide every year. The most common organs affected are the breast, colon, and prostate. In 2018, there were 10,518 cancer deaths in Saudi Arabia. According to the Saudi tumor registry, the prevalence of brain tumors (mainly brain cancers) is relatively low compared with other regions around the world.

Primary central nervous system (CNS) tumors are a diverse group of tumors originating in the brain and spinal cord. More than 90% of these tumors arise in the brain and the remainder occur in the meninges, spinal cord, cranial nerves, and the surrounding structures. From the 1970s onwards, the diagnosis of CNS tumors has increased worldwide, with age-standardized incidence rates ranging from 4.3 to 18.6 per 100,000 per year. The highest incidence of CNS tumors is in northern Europe, followed by the United States, Canada, and Australia [1].

CNS tumors have been categorized into different histological groups by the World Health Organization (WHO) [2,3]. Unlike most other tumor types, CNS tumors are not staged; instead, they are classified as malignant or benign tumors based on a grading system, in which grade I/II tumors are considered “low grade-benign,” whereas grade III/IV tumors are considered “high grademalignant.” Given the differences in incidence rates across geographical locations and according to sex, there is a need to understand the etiology and risk factors of CNS tumors. The most common primary CNS tumor worldwide is glioma. The incidence of glioma is the highest in Australia, followed by Western Europe and North America [4]. The most common subtype is glioblastoma. The prevalence of glioblastoma in the United States in 2010 was estimated to be 47.6 per 100,000 [5]. In East Asia and Southern Europe, malignant meningioma is the most common malignant CNS tumor.

According to the Saudi cancer registry, the prevalence of brain cancers is relatively low in Saudi Arabia compared with other regions of the world. The most common malignant CNS tumor in Saudi Arabia is metastatic carcinoma followed by high-grade gliomas. Several studies found that the incidence of malignant CNS tumors is highest in males and in adults older than 40 years old [2], while benign tumors are more common in females [4]. In the pediatric population, the incidence of medulloblastoma is highest in Southern Europe and Eastern Europe, whereas astrocytic tumors are most common in the United States and Canada. There is no available data registry of CNS tumors in children in Saudi Arabia.

An expanded retrospective analysis of CNS tumors has never been conducted in Saudi Arabia. Only a single constrained retrospective analysis of cancer incidence was performed in the Eastern population of Saudi Arabia, and another general meta-analysis was performed that estimated brain cancer to account for 9.5% of all cancer diagnoses [6]. In the current study, we performed a retrospective analysis of CNS tumors in the Western Province of Saudi Arabia in the period 2014-2019. This study focused on generating a fine-grained epidemiological matrix, including age, sex, tumor location, histological subtype, genetic characteristics, and recurrence status. To our knowledge, this analysis is the first data registry of CNS tumors in Saudi Arabia that involves case distribution and an epidemiological matrix.

## MATERIALS AND METHODS

### Study design and sample

A cross-sectional study was performed using secondary data obtained from 3 out of 6 tertiary governmental hospitals affiliated with neuroscience centers, which receive cases of brain tumors: King Abdulaziz University (KAU) Hospital, King Abdulaziz Medical City in Jeddah (KAMC) and King Faisal Specialist Hospital and Research Centers in Jeddah (KFSHRC). Clinical data between 2014 and 2019, including age, sex, tumor location, histological classification, type of surgery, laboratory findings, and recurrence rates, were obtained. The dataset included 663 patients in adult and pediatric populations diagnosed with primary CNS tumors in the Western Province of Saudi Arabia.

### Methodology

All cases were coded following the International Classification of Diseases for Oncology, third edition. The histological codes of tumors were then matched and grouped according to the categorization highlighted in the WHO 2016 classification of CNS tumors. Any tumors that lacked histological confirmation were removed from the dataset. Based on the diagnosed cases, the investigators identified primary CNS tumors under different categories. Patients under the age of 18 at the time of diagnosis were placed within the pediatric group. Although some cases had no genetic investigation, they were included in the analysis. IBM SPSS version 24 (IBM Corp., Armonk, NY, USA) and Microsoft Excel (Microsoft, Redmond, WA, USA) were used for the statistical analysis. A p-value < 0.01 was considered to indicate statistical significance.

### Ethics statement

The study was ethically approved by the National Biomedical Ethics Committee at KAU (HA-02-J-008) (Reference 190-19). This ethical approval was linked to additional approvals obtained from 2 medical centers (KAMC and KFSHRC). Following ethical approval, data on primary CNS tumors from the tumor registry were shared with the investigators. Ethics approval mandated that the researchers followed the procedures stipulated by “Good Clinical Practice” and the “Collaborative Institutional Training Initiative on Social and Behavioral Research.”

## RESULTS

Data on 663 patients with CNS tumors, diagnosed between 2014-2019, were retrieved from hospital records. As the tumor cases were collected over a 5-year period, some values of variables were inevitably missing. To overcome bias in the data analysis, these cases were excluded from the study, although cases with missing genetic results were included. The crude incidence rate was also difficult to calculate because there was no annual data registry from each hospital.

**Figure 1.**
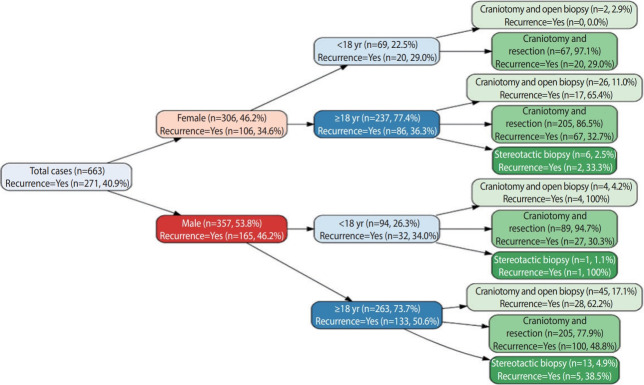
Distribution of central nervous system tumors by sex, age, type of surgical procedure, and recurrence rate.

The cases enrolled in this study were from the local and expatriate populations. The cases included both adult and pediatric patients. Five hundred cases (75.4%) were adults, and 163 cases (24.6%) were children aged 18 or under, with a male-to-female ratio of 1.16. The mean age at diagnosis was 38.0± 22.6 years (Figure 1 and Table 1). Approximately 43.1% of the cases were in the 25-54 years age group when diagnosed and 11.8% of the cases were diagnosed with a CNS tumor after the age of 65 years (Table 2).

**Figure 2.**
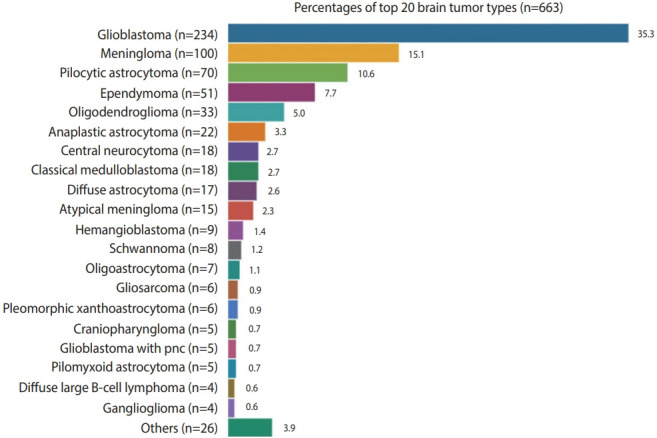
The incidence of central nervous system tumors in the Western Province of Saudi Arabia in the period 2014–2019.

The supratentorium was the most common location, with 515 cases (77.7%), followed by 144 cases (21.7%) in the infratentorium (posterior fossa, cerebellum, and fourth ventricle). The most predominant supratentorial site was the frontal lobe (26.4%, n= 175), in which intra-axial tumors (glioblastoma followed by oligodendroglioma) were the most commonly diagnosed tumors. Meningioma was the most common benign extra-axial tumor in the frontal lobe. Pilocytic astrocytoma (n = 70, 10.6%) was the most common CNS tumors in the posterior fossa and predominantly occurred in pediatric patients. Overall, glioblastoma was the most common CNS tumor (n= 234, 35.3%) followed by meningioma (n= 100, 15.1%). Hence, low-grade tumors (WHO I/II) were more predominant in the CNS than high-grade tumors (WHO III/IV). Diffuse oligodendroglial tumors accounted for 5.0% of all glial tumors, whereas diffuse astrocytic tumors accounted for 2.6%. Because glioblastoma with a primitive neuronal component (PNC) is rare and unique, it was categorized separately and accounted for 0% of all CNS tumors. Other tumors included dysembryoplastic neuroectodermal tumor, pineal gland tumors, subependymal giant cell astrocytoma, and choroid plexus papilloma, which together accounted for 3.9% of all CNS tumors (Figure 2).

The patients enrolled in this analysis commonly presented with headache (44.9%, n= 298). The next most common symptom was a focal neurological deficit (19.9%, n= 132). Most adult cases who presented with seizures or epilepsy had glioblastoma or oligodendroglioma, while most pediatric cases who presented with seizure had either cortical pilocytic astrocytoma or ependymoma. Of the patients who presented with visual impairment, 5.0% of the total patients, enrolled in this study, were presented with visual impairment and their tumors were sellar, suprasellar, or optic meningiomas.

**Figure 3.**
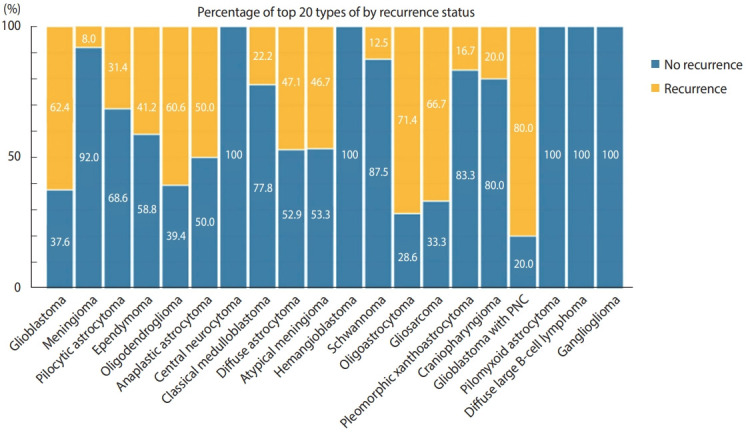
The recurrence status of the most common central nervous system tumors in the Western Province of Saudi Arabia.

Approximately 85.4% (n= 566) of the patients underwent surgical craniotomy and resection of the tumor, while 97 cases only underwent biopsy. The cases diagnosed via a brain biopsy were mostly high-grade gliomas and diffuse large B-cell lymphomas. The recurrence rate of CNS tumors was estimated to be approximately 41%. The most recurrent tumors were classical glioblastoma (63.5%, n = 155), followed by glioblastoma with PNC and gliosarcoma. The recurrence rate was close to 0% in cases of hemangioblastoma, schwannoma, central neurocytoma, ganglioglioma and pilocytic/pilomyxoid astrocytoma. In the pediatric group, medulloblastoma showed low recurrence rate of 22.2%. This was either because of the low volume of samples or because the patients received good management. Figure 3 summarizes the recurrence rate of the top 20 CNS tumor types.

Our results also revealed that glioma represented the most common primary CNS tumor in the Western Province of Saudi Arabia, accounting for 62.3% of cases, with a male-to-female ratio of 1.5 (Table 3). Glioblastoma (WHO-IV glioma) was more prevalent in male patients (64.3%, n = 157) than in female patients (35.7%. n= 87). Oligodendroglioma was the only grade II glioma that was slightly more prevalent in females than males, with a difference of only 2 cases. There was a significant difference in the age distribution between adult and pediatric gliomas (p< 0.01). CNS gliomas were more prevalent in adult patients (79.2%, n= 327) than in pediatric patients (20.8%, n= 86). Glioblastoma was the predominant CNS glioma in adult patients, 95.5% (233/344) of the glioblastoma found in adult. The glioma that showed the strongest predilection for pediatric patients was pilocytic astrocytoma (83.3%. n= 60), which was mainly found in the posterior fossa. Approximately 8% of oligodendroglioma cases were children. There was a significant difference in tumor location among all CNS gliomas (p< 0.001). The frontoparietal area was the most predominant location for glioblastoma (70-87%). Oligodendroglioma was predominantly observed in the frontal lobe (21/34 cases; 61.8%).

The most common clinical features associated with CNS gliomas are headache and focal neurological deficit, which commonly accompany glioblastoma and cortical pilocytic astrocytoma. Epilepsy was frequently associated with glioblastoma (40.1%, n= 97) and oligodendroglioma (58.3%, n= 21). Our results also showed that most high-grade gliomas were enhanced on magnetic resonance imaging (MRI) after gadolinium contrast. Oligodendrogliomas and pilocytic astrocytomas also showed partial enhancement on MRI.

The methylation status of methylguanine methyl transferase (*MGMT*) was not routinely assessed in all CNS gliomas. In the current data, we found that *MGMT* was methylated in 26/31 cases of glioblastoma that were tested. Other CNS gliomas, such as pilocytic astrocytoma or oligodendroglioma, did not show *MGMT* methylation. Because isocitrate dehydrogenase 1 (*IDH1*) testing is not routinely performed in most medical centers, few tumors were tested for *IDH1* mutation via immunohistochemistry or gene sequencing. Therefore, out of the 104 glioblastoma cases, 62 (59.0%) were wild-type for *IDH1* and 42 (40.2%) carried an *IDH1* mutation.

**Figure 4.**
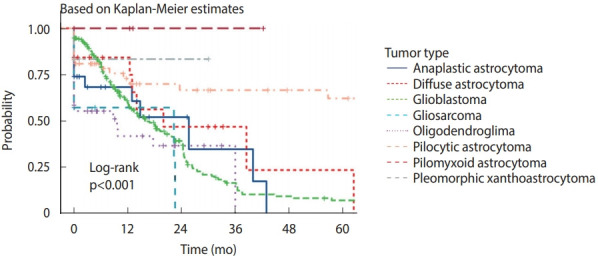
Recurrence interval for common central nervous system gliomas in the Western Province of Saudi Arabia.

Most of the CNS gliomas (81.6%) underwent complete surgical resection. Non-resection of tumors was because of surgical inaccessibility or poor patient health status. In 10 cases of non-resected glioblastoma, the tumor was located in a subcortical structure (basal ganglia or thalamus). There was a significant difference in recurrence rate among CNS gliomas (p< 0.001). The recurrence rate was the highest among glioblastoma patients (63.5%) and the lowest was among pilocytic astrocytomas (69.4%) (Figure 4).

## DISCUSSION

The highest incidence of CNS tumors is in Northern Europe, followed by the United States, Canada, and Australia [1]. Given the difference in incidence rates across geographical locations and according to sex, there is a need to understand the etiology and risk factors of CNS tumors [2,3]. The most common CNS tumors were grade VI, which was reinforced by the high number of gliomas recorded in the limited registry. However, the second most common tumor was benign meningioma (WHO grade I). With health insurance and government-funded hospital treatment, the likelihood of cases going undiagnosed has decreased.

Barnholtz-Sloan et al. [4] found that the global incidence of malignant CNS tumors was highest in male patients and adults older than 40, while benign tumors were found to be more common in female patients. Among children less than 15 years old, the highest incidence of CNS tumors was in children aged between 0-4 years. In the Western Province of Saudi Arabia, the mean age at diagnosis of all CNS tumors in males and females was 38 years. This is different from the United States, where the mean age is 59 years, but is similar to the United Arab Emirates, where the mean age is 33.4 years [7]. The predominant age at CNS tumor diagnosis was between 25-54 years. There was no significant difference in the sex distribution of all CNS tumors in the Western Province of Saudi Arabia (male-to-female ratio: 1.16). The highest incidence of CNS tumors was in children aged between 0-14 years.

The incidence of gliomas is highest in Australia, followed by Western Europe and North America [4]. The most common subtype is glioblastoma. Although most CNS tumors diagnosed in the United States are benign, the prevalence of glioblastomas in 2010 was estimated to be 47.6 per 100,000 (103,634 total cases) [5]. In the period from 2014 to 2019 in the Western Province of Saudi Arabia, the most commonly diagnosed CNS tumors were also gliomas, mainly glioblastoma (35.3%). Most of these glioblastomas were supratentorial (frontoparietal area), *IDH1* wild-type, *MGMT* non-methylated, and associated with poor outcomes and a high recurrence rate [8,9]. Most common pediatric tumors diagnosed in the Western Province of Saudi Arabia were pilocytic astrocytomas that were predominantly found in the posterior fossa. In contrast, diffuse astrocytoma and embryonic tumors are commonly diagnosed in pediatric patients in the United Arab Emirates [7].

After diagnosis with a malignant CNS tumor, the 1-year relative survival rate was 56.6%, with a 5-year relative survival rate of 32.1% [4]. Mortality caused by malignant CNS tumors also varies across the globe [1]. The mortality is highest in Northern Europe. In the Western Province of Saudi Arabia, most CNS gliomas (81.6%) underwent complete surgical resection. Non-resection of tumors was because of surgical inaccessibility or poor patient health status. There was also a significant difference in the recurrence rate among CNS gliomas (p< 0.001). The recurrence rate was highest among glioblastoma patients and lowest among those with pilocytic astrocytoma (Figure 4).

The only limitation acknowledged in this study is a lack of other specific epidemiological parameters (such as mortality rate) and non-registered cases from non-tertiary hospitals. However, this study is novel in that it presents the corresponding data from this region for the first time, which included age, sex, location and histological types, as well as the distribution of brain tumors in the Western Province of Saudi Arabia, which is notable for having higher levels of immigrants and expatriates than other local regions.

In conclusion, the incidence, prevalence, management, and recurrence of CNS tumors vary according to histological type, age at diagnosis, sex, and available treatments. A data registry of CNS tumors for each hospital is encouraged. Further research should be performed to identify potential risk factors for CNS tumors in Saudi Arabia. Genome technology (including genomic, epigenomic, transcriptomic, proteomic, and metabolomic approaches) is important in the healthcare system and provides an opportunity to identify relationships between the incidence rate and risk factors. Further large, multicenter epidemiological studies as well as well-annotated omics datasets are important for advancing CNS tumor research.

## Figures and Tables

**Table 1.** Descriptive distribution of CNS tumors in the Western Province of Saudi Arabia (n=663)

Characteristics	n (%)
Sex	
Female	306 (46.2)
Male	357 (53.8)
Age (yr)	
<18	163 (24.6)
≥ 18	500 (75.4)
Mean±SD [range]	38.0±22.6 [1.0-95.0]
Clinical presentation	
Cranial nerve palsy	17 (2.6)
Focal neurological deficit	132 (19.9)
Headache	298 (44.9)
Other	60 (9.0)
Seizure	123 (18.6)
Visual impairment	33 (5.0)
Type of surgery	
Craniotomy and open biopsy	77 (11.6)
Craniotomy and tumor resection	566 (85.4)
Stereotactic biopsy	20 (3.0)
Tumor location	
Third ventricle	2 (0.3)
Fourth ventricle	4 (0.6)
Basal ganglia	4 (0.6)
Brainstem	8 (1.2)
Cerebellum	15 (2.3)
Cerebellopontine angle	18 (2.7)
Intradural extramedullary	2 (0.3)
Frontal	175 (26.4)
Interhemispheric	3 (0.5)
Intramedullary	2 (0.3)
Jugular foramen	1 (0.2)
Lateral ventricle	32 (4.8)
Occipital	22 (3.3)
Olfactory groove	5 (0.8)
Optic chiasm	3 (0.5)
Optic nerve	5 (0.8)
Parasagittal	11 (1.7)
Parietal	97 (14.6)
Pineal region	4 (0.6)
Planum	4 (0.6)
Posterior fossa	98 (14.8)
Sellar	1 (0.2)
Sphenoid wing	12 (1.8)
Suprasellar	21 (3.2)
Temporal	99 (14.9)
Thalamic	15 (2.3)
Recurrence status	
No recurrence	392 (59.1)
Recurrence	271 (40.9)

CNS, central nervous system; SD, standard deviation.

**Table 2.** Age distribution of CNS tumor patients in the Western Province of Saudi Arabia

Age at diagnosis	n (%)
<14	144 (21.7)
15-24	52 (7.8)
25-54	286 (43.1)
55-64	103 (15.5)
≥ 65	78 (11.8)
Total	663 (100)

CNS, central nervous system.

**Table 3.** Descriptive and statistical distribution of CNS gliomas in the Western Province of Saudi Arabia

Variables		Anaplastic astrocytoma	Diffuse astrocytoma	Glioblastoma	Gliosarcoma	Oligodendroglioma	Pilocytic astrocytoma	Pilomyxoid astrocytoma	Pleomorphic xanthoastrocytoma	Total	p-value
Sex											0.354
	Female	10 (43.5)	10 (50.0)	87 (35.7)	2 (28.6)	19 (52.8)	31 (43.1)	2 (40.0)	4 (66.7)	165 (40.0)	
	Male	13 (56.5)	10 (50.0)	157 (64.3)	5 (71.4)	17 (47.2)	41 (56.9)	3 (60.0)	2 (33.3)	248 (60.0)	
Age (yr)											< 0.001^1^
	<18	1 (4.3)	2 (10.0)	11 (4.5)	1 (14.3)	3 (8.3)	60 (83.3)	4 (80.0)	4 (66.7)	86 (20.8)	
	≥18	22 (95.7)	18 (90.0)	233 (95.5)	6 (85.7)	33 (91.7)	12 (16.7)	1 (20.0)	2 (33.3)	327 (79.2)	
Tumor location											< 0.001^1^
	Brainstem	0 (0.0)	1 (6.2)	1 (0.4)	0 (0.0)	0 (0.0)	4 (16.7)	0 (0.0)	0 (0.0)	6 (1.8)	
	Cerebellum	0 (0.0)	0 (0.0)	3 (1.3)	0 (0.0)	0 (0.0)	3 (12.5)	0 (0.0)	1 (16.7)	7 (2.0)	
	Frontal	13 (59.1)	6 (37.5)	78 (33.8)	3 (42.9)	21 (61.8)	4 (16.7)	0 (0.0)	0 (0.0)	125 (36.5)	
	Occipital	1 (4.5)	0 (0.0)	12 (5.2)	1 (14.3)	1 (2.9)	1 (4.2)	0 (0.0)	0 (0.0)	16 (4.7)	
	Parietal	3 (13.6)	3 (18.8)	70 (30.3)	2 (28.6)	4 (11.8)	3 (12.5)	0 (0.0)	3 (50.0)	88 (25.7)	
	Temporal	3 (13.6)	5 (31.2)	61 (26.4)	1 (14.3)	7 (20.6)	5 (20.8)	1 (50.0)	2 (33.3)	85 (24.9)	
	Thalamic	2 (9.1)	1 (6.2)	6 (2.6)	0 (0.0)	1 (2.9)	4 (16.7)	1 (50.0)	0 (0.0)	15 (4.4)	
Clinical presentation											< 0.001^1^
	Cranial nerve palsy	0 (0.0)	1 (5.0)	0 (0.0)	0 (0.0)	0 (0.0)	6 (8.5)	0 (0.0)	0 (0.0)	7 (1.7)	
	Focal neurological deficit	5 (22.7)	4 (20.0)	60 (24.8)	0 (0.0)	2 (5.6)	11 (15.5)	1 (20.0)	1 (16.7)	84 (20.5)	
	Headache	10 (45.5)	6 (30.0)	97 (40.1)	5 (71.4)	11 (30.6)	32 (45.1)	3 (60.0)	2 (33.3)	166 (40.6)	
	Other	2 (9.1)	2 (10.0)	25 (10.3)	1 (14.3)	2 (5.6)	7 (9.9)	0 (0.0)	1 (16.7)	40 (9.8)	
	Seizure	5 (22.7)	6 (30.0)	54 (22.3)	1 (14.3)	21 (58.3)	10 (14.1)	0 (0.0)	2 (33.3)	99 (24.2)	
	Visual impairment	0 (0.0)	1 (5.0)	6 (2.5)	0 (0.0)	0 (0.0)	5 (7.0)	1 (20.0)	0 (0.0)	13 (3.2)	
Radiological findings											< 0.001^1^
	Calcification	0 (0.0)	0 (0.0)	0 (0.0)	0 (0.0)	1 (3.0)	1 (1.4)	0 (0.0)	0 (0.0)	2 (0.5)	
	Enhanced no edema	3 (13.6)	1 (5.6)	18 (8.1)	1 (16.7)	3 (9.1)	18 (25.0)	2 (40.0)	0 (0.0)	46 (12.0)	
	Enhanced with edema	16 (72.7)	8 (44.4)	190 (86.0)	5 (83.3)	22 (66.7)	42 (58.3)	2 (40.0)	4 (80.0)	289 (75.7)	
	Non-enhanced no edema	1 (4.5)	4 (22.2)	0 (0.0)	0 (0.0)	2 (6.1)	7 (9.7)	1 (20.0)	0 (0.0)	15 (3.9)	
	Non-enhanced with edema	2 (9.1)	5 (27.8)	13 (5.9)	0 (0.0)	5 (15.2)	4 (5.6)	0 (0.0)	1 (20.0)	30 (7.9)	
*MGMT* methylation status											< 0.001^1^
	*MGMT* methylation			26 (83.9)						26 (83.9)	
	No *MGMT* methylation			5 (16.1)						5 (16.1)	
*IDH1* status											< 0.001^1^
	*IDH1* mutant			42 (40.2)						42 (40.2)	
	*IDH1* wild-type			62 (59.0)						62 (59.0)	
Resection											0.005
	No	8 (34.8)	2 (10.0)	57 (23.4)	1 (14.3)	2 (5.6)	6 (8.3)	0 (0.0)	0 (0.0)	76 (18.4)	
	Yes	15 (65.2)	18 (90.0)	187 (76.6)	6 (85.7)	34 (94.4)	66 (91.7)	5 (100)	6 (100)	337 (81.6)	
Recurrence											< 0.001^1^
	No recurrence	11 (47.8)	11 (55.0)	89 (36.5)	2 (28.6)	15 (41.7)	50 (69.4)	5 (100)	5 (83.3)	188 (45.5)	
	Recurrence	12 (52.2)	9 (45.0)	155 (63.5)	5 (71.4)	21 (58.3)	22 (30.6)	0 (0.0)	1 (16.7)	225 (54.5)	

Values are presented as number (%). CNS, central nervous system; MGMT, methylguanine methyl transferase; IDH1, isocitrate dehydrogenase 1.

1 Pearson’s chi-squared test.
